# Expression of Toll-Like Receptors 2 and 4 and Related Cytokines in Patients with Hepatic Cystic and Alveolar Echinococcosis

**DOI:** 10.1155/2015/632760

**Published:** 2015-11-09

**Authors:** Tuerhongjiang Tuxun, Hai-Zhang Ma, Shadike Apaer, Heng Zhang, Amina Aierken, Yu-Peng Li, Ren-Yong Lin, Jin-Ming Zhao, Jin-Hui Zhang, Hao Wen

**Affiliations:** ^1^State Key Laboratory Incubation Base of Xinjiang Major Diseases Research and Xinjiang Key Laboratory of Echinococcosis, First Affiliated Hospital of Xinjiang Medical University, Ürümqi 830054, China; ^2^Department of Liver and Laparoscopic Surgery, Digestive and Vascular Centre, First Affiliated Hospital of Xinjiang Medical University, Ürümqi 830054, China; ^3^Department of General Surgery, Qilu Hospital, Shandong 250000, China; ^4^Department of Ultrasonography, First Affiliated Hospital of Xinjiang Medical University, Ürümqi 830054, China

## Abstract

Several studies have demonstrated the important role of Toll-like receptors in various parasitic infections. This study aims to explore expression of Toll-like receptors (TLRs) and related cytokines in patients with human cystic echinococcosis (CE) and alveolar echinococcosis (AE). 78 subjects including AE group (*N* = 28), CE group (*N* = 22), and healthy controls (HC, *N* = 28) were enrolled in this study. The mRNA expression levels of TLR2 and TLR4 in blood and hepatic tissue and plasma levels related cytokines were detected by using ELISA. Median levels of TLR2 mRNA in AE and CE groups were significantly elevated as compared with that in healthy control group. Median levels of TLR4 expression were increased in AE and CE. Plasma concentration levels of IL-5, IL-6, and IL-10 were slightly increased in AE and CE groups compared with those in HC group with no statistical differences (*p* > 0.05). The IL-23 concentration levels were significantly higher in AE and CE groups than that in HC subjects with statistical significance. The increased expression of TLR2 and IL-23 might play a potential role in modulating tissue infiltrative growth of the parasite and its persistence in the human host.

## 1. Introduction

Human echinococcosis, also known as hydatid disease, is one of the geographically widely distributed neglected tropical diseases [1]. Human cystic echinococcosis (CE) and alveolar echinococcosis (AE) are two of the most commonly seen subtypes, respectively, caused by the larval stages of* Echinococcus granulosus* (*E. granulosus*) and* Echinococcus multilocularis* (*E. multilocularis*). Both CE and AE are commonly seen in pastoral and/or semipastoral area in China, Central Asia, Middle East, South America, and some part of Europe and continue to be a major public health issue [2].

The mainly targeted organ during the infection is the liver by about 70% in CE and 95% in AE. However, different organs could be affected by both types including lung, spleen, brain, bone, and even orbit. The parasite and host interaction is still a major interest of research, and it is widely acknowledged that the parasite plays an active role, to some extent, in modulating and evading host's immune system for successful survival. Previous studies have shown that parasite and its secretory and/or excretory molecules may initiate an appropriate polarized T helper (Th) cell response that will allow the parasite to escape cytotoxic attacks from the immune system and survive in the host [3]. Recognition of the parasite and its components is known to develop through pattern recognition receptors (PRRs), including Toll-like receptors (TLRs). To date, thirteen mammalian TLRs have been described, with ten expressed in humans, each responsible for the recognition of distinct, invariant structures, not expressed by the host, and known as pathogen-associated molecular patterns (PAMPs) [4]. Recently, TLRs have been demonstrated to be essential for activation of immune cells, including macrophages and dendritic cells, through the recognition of microbial and parasitic components including lipopolysaccharide (LPS) from Gram-negative bacteria, lipoprotein acid, lipoproteins produced by all bacterial pathogens, and peptidoglycan [5]. Several studies have demonstrated the important role of TLRs in various parasitic infections [6].

However, very little is known about the alteration pattern and possible role of TLRs in both types of echinococcosis, especially in alveolar echinococcosis to date. In this study, we have assessed the expression and discussed potential role of TLR2 and TLR4 in CE and AE patients and compared their expressions, circulating cytokines, and correlations, if any, in patients and healthy controls.

## 2. Materials and Methods

### 2.1. Subjects

78 subjects were enrolled in this study including 28 consecutive AE patients, 22 consecutive CE patients, and 28 gender- and age-matched healthy controls recruited from healthy staff members of our department. There was no significant difference in the baseline characteristics between the three groups.* E. granulosus* or* E. multilocularis* infestations were confirmed by postoperative pathological examination. The classification of CE and AE was staged according to the WHO Informal Working Group on Echinococcosis (WHO-IWGE) classification revised criteria [7]. No patient was treated with anti-inflammatory drugs such as nonsteroidal anti-inflammatory agents and corticosteroids. No patients had chronic inflammatory disease, cardiovascular disease, disseminated intravascular coagulation, advanced lung disease, renal failure, malignant disease, jaundice, or other infectious diseases (such as septicemia and pneumonia).

### 2.2. Preparation of Peripheral Blood Mononuclear Cells (PBMCs)

Blood samples were obtained from all healthy controls and patients on the following morning of the admission day. The samples were collected into collection tubes containing 0.2 mL sodium heparin. Plasma was obtained after centrifugation and stored at −20°C for the measurement of related cytokine levels. In addition, PBMCs were prepared by Ficoll-Histopaque (1.077 kg/L, Sigma-Aldrich, St. Louis, MO, USA) by density gradient centrifugation for analysis by real-time-polymerase chain reaction (qRT-PCR).

### 2.3. Surgery and Liver Tissue Samples

All resections of hepatic AE patients were performed via laparotomy. All surgical procedures were performed by three experienced hepatic surgeons. Experienced senior surgeons carried out all partial hepatic resections to guarantee at least two-centimeter safety margin. Frozen section examinations at the hepatic transection line were performed in all cases. All surgical specimens were reviewed by a senior pathologist. Clinical and pathologic staging were reassessed according to the World Health Organization Informal Working Group on Echinococcosis (WHO-IWGE) classification [8]. The specimens were sectioned for lesion, paralesion (hepatic tissue within one cm from the AE lesion), and normal liver tissues. The specimens had been snap-frozen in RNA later and liquid nitrogen for 15 seconds and immediately stored at −80°C.

### 2.4. RNA Extraction

For TLR2 and TLR4 mRNA gene expression analysis, samples of the PBMCs and liver were stored at −80°C until total RNA extraction. Total RNA was extracted using the Trizol reagent (Invitrogen, Carlsbad, CA, USA) according to the manufacturer's instructions, at a ratio of 1 mL for every 100 mg liver tissue. Tissue specimens with Trizol were ground using an electric Polytron homogenizer until their complete dissociation, after which 200 *μ*L chloroform was added, and the samples were manually homogenized for 15 s. Samples were then centrifuged for 15 min at 12,000 rcf at 4°C for 10 min, and then 500 *μ*L isopropanol was added. Samples were again homogenized and centrifuged at 7500 rcf for 5 min. The supernatant was discarded, and the precipitate was washed in 1 mL 75% ethanol followed by centrifugation again under the same conditions, and after discarding the supernatant, the pellet was dried for about 15 min and then resuspended in RNAase-free ultrapure water. RNA concentration and quantity were estimated from the optical density at 260 and 280 nm and RNA purity was estimated using 260/280 ratio 1.8 to 2.0. RNAs were converted to cDNA using RevertAid Reverse Transcriptase (Thermo Scientific) and oligo (dT) 18 primers and were stored at −20°C until analysis.

### 2.5. Real-Time Fluorescent Quantitative Reverse-Transcription Polymerase Chain Reaction (qRT-PCR)

TLR2 and TLR4 mRNA expressions were all determined by using a commercial QuantiFast SYBR Green PCR Kit (QIAGEN, Germany) according to the manufacturer's instructions. The primers were synthesized by Shenggong Biotech (Shanghai, China) as follows: TLR2: forward, 5′-GGCATGTGCTGTGCTCTGTT-3′, reverse, 5′-GCTTTCCTGGGCTTCCTTTT-3′; TLR4: forward, 5′-TGAGCAGGTCTAGGGTGATTGAAC-3′, reverse, 5′-ATGCGGACACACACACTTTCAAATA-3′; and GAPDH: forward, 5′-GCACCGTCAAGGCTGAGAAC-3′, reverse, 5′-TGGTGAAGACGCCAGTGGA-3′. GAPDH was analyzed as an internal control and all target genes were normalized to GAPDH. The quantitative PCR analyses of the data were performed using SYBR Green program on i-Q5.0 real-time PCR system (Bio-Rad, Foster City, CA, USA). The relative amounts of PCR product were determined using the relative standard curve method. The results were expressed in terms of relative TLR mRNA quantification. RNA expression level fold changes were calculated as described by the SYBR Green I protocol.

### 2.6. Enzyme-Linked Immunosorbent Assay (ELISA)

Plasma IFN-*γ* (assay sensitivity was 4 pg/mL), IL-23 (assay sensitivity was 4 pg/mL), IL-10 (assay sensitivity was 2 pg/mL), IL-5 (assay sensitivity was 2 pg/mL), and IL-6 (assay sensitivity was 4 pg/mL) concentrations were determined from 50 patients and 28 healthy controls by ELISA using a commercial human ELISA kit (eBioscience, San Diego, CA, USA), according to the manufacturer's instructions. Cytokine concentrations were calculated by using the mean optical density of two wells and comparison with a standard curve.

### 2.7. Statistical Analysis

Values were expressed as median (IQR) in text and figures. The nonparametric Kruskal-Wallis *H* test followed by Mann-Whitney *U* test and nonparametric two-related-sample tests (Wilcoxon signed rank test) were applied for multiple comparisons between three different independent groups and the relative TLR2 and TLR4 expression levels in liver tissues. Spearman's correlation analysis was used as a test of correlation between two continuous variables. Correlations were determined by Spearman correlation coefficients. A *p* value less than or equivalent to 0.05 (*p* value of two-related-sample test was 0.05/3) was considered to be statistically significant and considered as extremely significant (*∗∗∗*) (*p* < 0.001); highly significant (*∗∗*) (*p* < 0.01); significant (*∗*) (*p* < 0.05); and not significant (*p* > 0.05). All statistical analyses were performed by using SPSS statistics software version 17.0, and all graphs were presented using GraphPad Prism software version 5.0.

## 3. Results

### 3.1. Basic Clinical Characteristics of Patients

Age, gender, ethnic groups, diameters of lesions, and other base lines are listed in Table 1. There were no significant differences between the three groups.

### 3.2. Levels of TLR2 and TLR4 mRNA Expression in PBMCs

**Figure 1:**
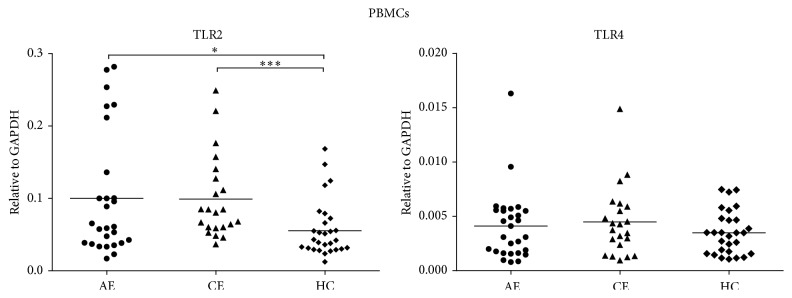
RT-qPCR analyses of TLR2 and TLR4 mRNA expressions in different groups. The relative expressions of TLR2 and TLR4 were calculated, normalized to GAPDH mRNA. The RT-qPCR data were analyzed using 2^−Δct^ method. Significantly increased TLR2 mRNA expression levels were observed in AE and CE patients when compared with HC subjects (*p* < 0.05, *p* < 0.01, resp.). No statistical differences were found in the relative TLR4 mRNA expressions between three groups (*p* > 0.05).

We have detected the relative expression levels of TLR2 and TLR4 mRNA in PBMCs. As shown in Figure 1, median levels of TLR2 mRNA in AE (0.0595, interquartile range (IQR): 0.0385 to 0.1272) and CE (0.0823; IQR: 0.0596 to 0.1304) groups were significantly elevated as compared with that in healthy control group (0.0436; IQR: 0.0305 to 0.0709; *p* = 0.027, *p* = 0.0006). No statistical difference was found between AE and CE groups (*p* > 0.05). Median levels of TLR4 expression were increased in AE (0.0036; IQR: 0.0017 to 0.0056) and CE (0.0040; IQR: 0.0021 to 0.0060) groups when compared to HC (0.0036; IQR: 0.0017 to 0.0048) group; however, no significant difference was found between them (*p* > 0.05).

### 3.3. Percentage of Eosinophil Cells and Its Correlations with mRNA Levels of TLR2 and TLR4 in Peripheral Blood

**Figure 2:**
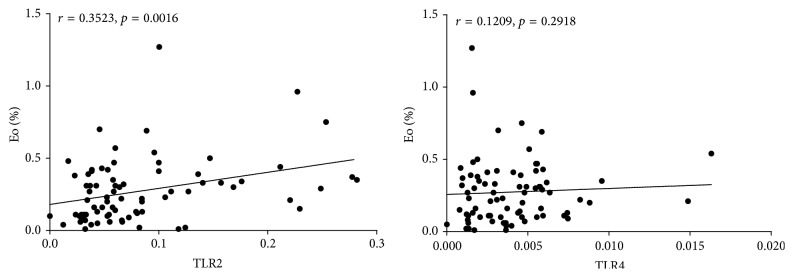
Correlations between mRNA levels of TLR2 and TLR4 and eosinophil percentage in peripheral blood. Spearman correlation coefficients indicated a significant positive correlation between the relative TLR2 mRNA levels and eosinophil percentage (*r* = 0.3523, *p* < 0.01). There were also linear correlations between TLR4 mRNA levels and eosinophil percentage, however, with no statistical significance (*p* > 0.05).

The median percentage of eosinophil cells in AE (0.41, IQR: 0.32 to 0.48) and CE (0.27; IQR: 0.19 to 0.31) groups was significantly elevated as compared with that in healthy control group (0.10; IQR: 0.05 to 0.13; *p* < 0.01). Spearman correlation coefficients indicated a significant positive correlation between the relative TLR2 mRNA levels with eosinophil percentage (*r* = 0.3523, *p* < 0.01). There were linear correlations between relative TLR4 mRNA expression levels with eosinophil percentage, however, with no statistical significance (*p* > 0.05) as shown in Figure 2.

### 3.4. Concentration Levels of IL-5, IL-6, IL-10, IL-23, and IFN-*γ* in Plasma in Different Groups

**Figure 3:**
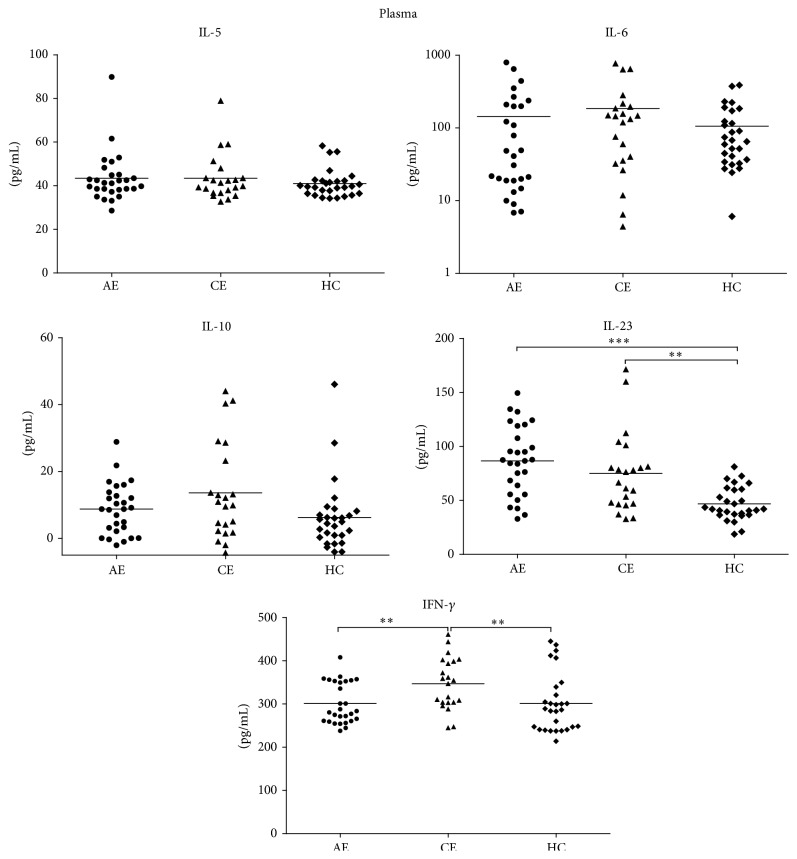
Concentration levels of cytokines in plasma from all subjects were detected using ELISA. Plasma concentration levels of IL-5, IL-6, and IL-10 were slightly increased in AE and CE groups compared with those in HC group with no statistical differences (*p* > 0.05). The IL-23 concentration levels were significantly higher in AE subjects as well as in CE subjects than these in HC subjects with statistical differences (*p* < 0.001, *p* < 0.01, resp.). However, no differences were observed between AE and CE groups (*p* > 0.05). Concentration levels of IFN-*γ* in CE subjects were markedly higher than those in HC subjects (*p* < 0.01); interestingly, decreased IFN-*γ* concentration levels in AE subjects were also observed when compared with CE subjects with statistical differences (*p* < 0.01).

Concentration levels of cytokines in plasma from all subjects were detected using ELISA technique. As shown in Figure 3, plasma concentration levels of IL-5, IL-6, and IL-10 were slightly increased in AE and CE groups compared with those in HC group with no statistical differences (*p* > 0.05). The IL-23 concentration levels were significantly higher in AE subjects as well as in CE subjects than that in HC subjects with statistical significance (*p* < 0.001, *p* < 0.01, resp.). However, no statistical difference was observed between AE and CE groups (*p* > 0.05). Concentration levels of IFN-*γ* in CE subjects (350.4395; IQR: 303.1556–399.1135) were markedly higher than those in HC subjects (287.7075; IQR: 242.1812–334.6671) (*p* < 0.01). Interestingly, we also observed a decreased IFN-*γ* concentration level in AE subjects when compared to CE subjects with statistical differences (*p* < 0.01).

### 3.5. Levels of TLR2 and TLR4 mRNA Expression in Hepatic Tissues

**Figure 4:**
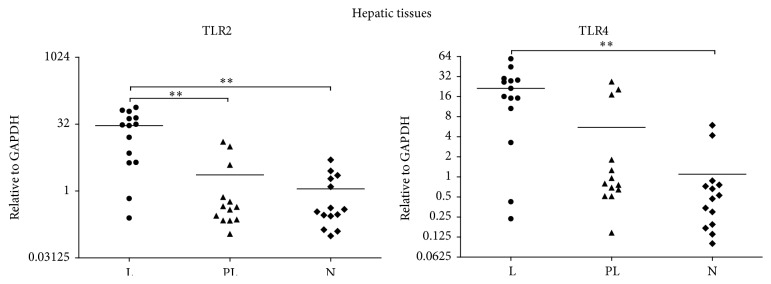
The relative expression levels of TLR2 and TLR4 mRNA in lesion, paralesion, and normal hepatic tissues by qRT-PCR. The expression levels of TLR2 mRNA in lesion tissues were markedly higher than those in paralesion and normal tissues (*p* = 0.004, *p* = 0.002, resp.), and there was no statistical significance between paralesion group and normal group (*p* > 0.05/3). TLR4 mRNA expression levels were markedly elevated in lesion tissues as compared with those in normal tissues (*p* = 0.002). Expression level of TLR4 mRNA is decreased in paralesion group as compared with lesion group and increased when comparing with normal tissues, although no statistical differences were found between them (*p* > 0.05/3). L: lesion; PL: paralesion; N: normal tissue.

The relative expression levels of TLR2 and TLR4 mRNA in lesion, paralesion, and normal hepatic tissues were detected and the results are shown in Figure 4. The expression levels of TLR2 mRNA in lesion tissues (30.1770; IQR: 4.3944 to 48.6075) were markedly higher than those in paralesion tissues (0.4409; IQR: 0.2231 to 2.6903; *p* = 0.004) and normal tissues (0.3645; IQR: 0.2388 to 1.9815; *p* = 0.002), and there was no statistical significance between paralesion group and normal group (*p* > 0.05/3). TLR4 mRNA expression levels were markedly elevated in lesion tissues (18.7271; IQR: 8.8038 to 28.7317) as compared with those in normal tissues (0.4988; IQR: 0.1873 to 0.7870; *p* = 0.002). Expression level of TLR4 mRNA is decreased in paralesion group (0.8690; IQR: 0.6083 to 8.4140) as compared with lesion group and increased when comparing with normal tissues, although no statistical differences were found between them (*p* > 0.05/3).

### 3.6. Correlations between mRNA Levels of TLR2 and TLR4 in PBMCs and the Concentration Levels of IL-10 and IL-23

**Figure 5:**
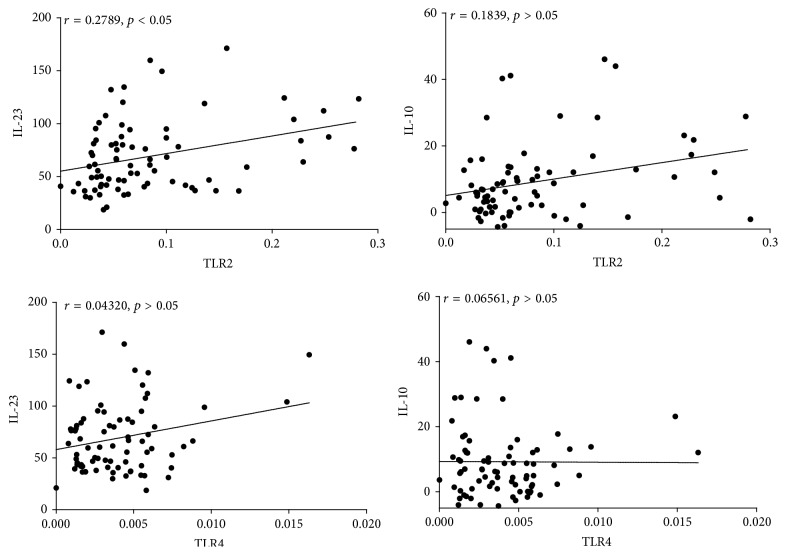
Correlations between mRNA levels of TLR2 and TLR4 in PBMCs and the concentration levels of IL-10 and IL-23. Spearman correlation coefficients indicated a significant positive correlation between the relative TLR2 mRNA levels and IL-23 concentration levels (*r* = 0.2789, *p* < 0.05). There were linear correlations between relative TLR2 mRNA expression levels and IL-10 concentration levels in PBMCs. There were also linear correlations between TLR4 mRNA levels and both IL-10 and IL-23 concentration levels in PBMCs, however, with no statistical significance (*p* > 0.05).

Spearman correlation coefficients indicated a significant positive correlation between the relative TLR2 mRNA levels and IL-23 concentration levels (*r* = 0.2789, *p* < 0.05). There were linear correlations between relative TLR2 mRNA expression levels and IL-10 concentration levels in PBMCs. There were also linear correlations between TLR4 mRNA levels and both IL-10 and IL-23 concentration levels in PBMCs, however, with no statistical significance (*p* > 0.05) as shown in Figure 5.

## 4. Discussion

Human CE and AE are caused, respectively, by the larval stage of* E. granulosus* and* E. multilocularis* [9, 10]. The egg hatched in the intestine will transform into oncosphere phase with the help of bile juice and penetrate through the intestinal wall and, thus, reside in the internal organ by following the portal blood stream. Both parasites can affect various host tissues and eventually develop as a fluid-filled cystic lesion in CE and a tumor-like infiltrative lesion in AE [11]. The liver is the most common targeted organ in CE (70%) and AE (95%), following liver, lung, and bone in some cases [12]. Despite being two different parasites belonging to the same family, they display an utterly distinct morphological and biobehavior which requests the different modality and results in different prognosis [13]. Like other parasitic infections, immune responses are now known to play a crucial role in the progress of disease development [14, 15]. During the long-term* E. granulosus*/*E. multilocularis* host interaction, the parasite has evolved a broad spectrum of abilities to modulate the host's immune system [16, 17]. Immunomodulation and, to some extent, immune tolerogenic role have been a great interest of researchers during infection both in human and in animal studies. Now, it is widely accepted that* E. granulosus/E. multilocularis* can actively interact with hosts' both innate and acquired immune system to maintain its survival with successful evasion from host's immune attacks [18–20].

Previous studies have shown that Th1- and Th2-type immune responses are important for clearance of the infection and are associated with the chronic and progressive course of disease [21]. However, in some cases of human echinococcosis, spontaneous healing of the disease was observed [22]. Such abortive cases are characterized by calcified parasite lesions suggesting the generation of immune responses which are able to limit parasite growth in humans.

As proposed by Janeway Jr. [23] two decades ago, the innate immune system senses microbial infection by using receptors that are predominantly expressed on sentinel cells referred to as “pattern recognition receptors (PRRs)” that recognize the molecular signature known as “pathogen-associated molecular patterns (PAMPs).” Toll like receptors are among the firstly discovered PRRs and have an important role in antigen recognition [24]. It is reported that helminthic excretory/secretory products such as* E. granulosus* antigen B,* E. multilocularis* protoscoleces, and soluble egg antigens (SEA, a soluble extract from* Schistosoma mansoni* eggs) inhibit the activation of DCs in response to classical Toll-like receptor (TLR) ligands such as lipopolysaccharide [25, 26]. Nevertheless, recent work has suggested that TLR4 and/or TLR2 play an important role in the recognition of helminthic products by DCs and macrophages and in the development of Th2 responses [26]. Recognition of parasite components is crucial for the initiation of an appropriate polarized T helper (Th) cell response that will allow the parasite to escape cytotoxic attacks from the immune system and survive in its host. Such recognition is thought to occur through interaction with PRRs, including TLRs [27]. The type of parasitic compound and of the PRR involved in their recognition will determine to a large extent the nature of the T cell polarization [28].

In this study, we have investigated the TLR2 and TLR4 expression in PBMCs in* E. granulosus* and* E. multilocularis* infected patients, as well as in fourteen AE patients' hepatic tissues. Our results showed significantly increased TLR2 mRNA expression levels in PBMCs in AE and CE patients compared to HC subjects. However, no statistical differences were found in the relative TLR4 mRNA expressions among three groups. Both TLR2 and TLR4 relative expression levels were slightly higher in AE than CE group; no statistical significance was shown between them.

The* E. granulosus/E. multilocularis* PAMPs might be recognized by different receptors and thus may lead to different immune response. Such increased recognition by TLR2 and TLR4 may play a critical role in initiating different immune response that might help the parasite to maintain its survival and continue the chronic inflammation. The discriminative expression of TLR2 and TLR4 between AE and CE, albeit with no significance, may partially explain the subtle immunotolerogenic differences between two species. Besides, the correlation study between eosinophil percentage in PBMCs and TLR2 expression showed a positive correlation with statistical significance. As eosinophil itself may express TLR2 [29], the TLR2 mRNA expressions might be partially attributed to the increased eosinophil percentage in PBMCs in AE and CE patients. Coherent to the findings in peripheral blood, both TLR2 and TLR4 mRNA expressions were higher in lesion compared to the normal tissue. This may be partially due to the infiltration of TLR expressing cells in the lesion. The relative high TLR2 and TLR4 niche may modulate antigen presenting and the parasite growth and granuloma formation process.

Since the up- or downregulated TLRs expressions lead to different T cell differentiation, thus, in this study, we have also detected Th1 related cytokine INF-*γ*, Th2 related cytokine IL-5, Treg related cytokine IL-10, and Th17 related cytokine IL-23 and, if any, their correlations with TLRs. The increased levels of IL-10 and IL-5, albeit with no statistical significance, might be a result of enhanced TLR2 and TLR4 recognition which skewed the naïve Th cells towards Treg and Th2 which is favored for parasite survival. Several studies have shown that helminthes can induce higher threshold ERK1/2 signaling through TLR intervention which increases IL-10 that plays a critical role in persistence of the infection [30, 31]. Both animal and human studies have shown the increased Treg cell immune response during both CE and AE infection [32, 33]. The increased expression of TLR2 and TLR4 by the Treg cells results in increased IL-10 which can play an immunotolerogenic role during the infection. Besides that, highly elevated IFN-*γ* levels were detected in CE compared to AE and HC. In line with this finding, Mezioug and Touil-Boukoffa [34] showed the increased levels of IFN-*γ* in Algerian CE patients and displayed a positive correlation with IL-17 levels and postulated the possible immune-protective role during the infection. However, it is not clear whether elevated IFN-*γ* levels are TLRs signaling or not, since IFN-*γ* may be activated independently on TLR signaling.

A growing body of evidence has shown the important role of IL-23 during various helminthic infections [35, 36]. However, very little was known about the alteration and potential role of IL-23 in echinococcosis. The current study showed that plasma levels of IL-23 in patients with CE and AE were significantly higher than those in HC (*p* < 0.05). Besides, the IL-23 level was positively related to the TLR2 mRNA expression in PBMCs. As a very important cytokine closely related to IL-23, cytokine IL-17 and mediated proinflammatory immune responses are shown to facilitate the tissue infiltrative growth of the parasite and its persistence in the human host [37]. IL-23 activates the adoptive and innate immune systems to produce IL-17A, IL-17F, IL-22, and TNF, all of which help to stimulate epithelial cells to produce antimicrobial factors. It is known that IL-23 mediates both protective and pathological functions [38]. Thus, the possible role and its mechanism require more detailed study in the future.

## 5. Conclusion

In conclusion, this study presented the first report on TLRs expression and its relationship with cytokine in patients with CE and AE. The increased expression of TLR2 and IL-23 might play a crucial role in modulating tissue infiltrative growth of the parasite and its persistence in the human host. A larger scale of the population and ongoing efforts should be made to identify the precise effect and mechanism of TLRs and related cytokines in immune tolerance and the progression of echinococcosis both in human and in animal models.

## Figures and Tables

**Table 1:** Basic clinical characteristics of patients with hepatic alveolar echinococcosis.

Characteristics	AE	CE	HC
	(*n* = 28)	(*n* = 22)	(*n* = 28)
Age (years)	39.5 (27.5–46.5)	34.0 (28.0–39.8)	30.0 (27.0–36.8)
Sex (male/female)	10 : 18	9 : 13	19 : 9
Location (right/left)	23 : 5	18 : 4	0
^#^Diameter of lesions (cm)	101.1 (80.0–223.9)	43.7 (22.2–67.7)	0
Ethnic group			
Tibet	6	3	5
Kazak	5	7	3
Han	6	4	7
Mongol	3	2	2
Uyghur	5	4	6
Other	3	2	5
Previous chemotherapy (%)	13 (46)	8 (36)	0 (0)

AE: alveolar echinococcosis; CE: cystic echinococcosis; HC: healthy controls.

^#^For those patients with multiple cysts, the average sizes were applied.
